# Breast Cancer in Megapolises of Kazakhstan: Epidemiological Assessment of Incidence and Mortality

**Published:** 2019-07

**Authors:** Nurbek IGISSINOV, Assem TOGUZBAYEVA, Botagoz TURDALIYEVA, Gulnur IGISSINOVA, Zarina BILYALOVA, Gulnur AKPOLATOVA, Murat VANSVANOV, Dinar TARZHANOVA, Akmaral ZHANTUREYEVA, Marina ZHANALIYEVA, Aigul ALMABAYEVA, Alikhan TAUTAYEV

**Affiliations:** 1. Department of Surgical Diseases Internship, Astana Medical University, Nur-Sultan, Kazakhstan; 2. Department of Science and Analytic, International High School of Medicine, Bishkek, Kyrgyzstan; 3. Central Asian Cancer Institute, Nur-Sultan, Kazakhstan; 4. Eurasian Institute for Cancer Research, Bishkek, Kyrgyzstan; 5. Department of Public Health, Asfendiyarov Kazakh National Medical University, Almaty, Kazakhstan; 6. Department of Science and Innovation, Kazakh Medical University of Continuing Education, Almaty, Kazakhstan; 7. Department of Oncology, Kazakh Medical University of Continuing Education, Almaty, Kazakhstan; 8. Department of General and Clinical Pharmacology, Astana Medical University, Nur-Sultan, Kazakhstan; 9. Department of Public Health with Nursing Course, Kazakh Medical University of Continuing Education, Almaty, Kazakhstan; 10 Department of Human Anatomy with Operative Surgery, Astana Medical University, Nur-Sultan, Kazakhstan

**Keywords:** Breast Cancer, Incidence, Mortality, Kazakhstan

## Abstract

**Background:**

Breast cancer is the most common malignant disease among the female population of Kazakhstan like in many developed countries of the world (Canada, UK, US, Western Europe), and it accounts for every 5^th^ tumor. We aimed to assess the epidemiological aspects of breast cancer incidence and mortality among Almaty and Astana (Now Nur-Sultan), Kazakhstan residents in 2009–2018.

**Methods:**

A retrospective study using modern descriptive and analytical methods of epidemiology was conducted to evaluate the breast cancer incidence and mortality in megapolises of Kazakhstan.

**Results:**

The average annual age-standardized incidence rate of breast cancer amounted to 61.9 00000 (95% CI=56.2–67.6) in Almaty and 61.2 00000 (95% CI=56.765.7) in Astana. The average age-standardized mortality was 19.2 00000 (95% CI=17.3–21.1) in Almaty and 19.3 00000 (95% CI=17.1–21.4) in Astana. The standardized incidence in the megapolises tended to increase (T_gr_=+0.8% in Almaty and T_gr_=+1.4% in Astana), while the mortality was decreasing (T_dec_=−4.2% in Almaty and T_dec_=−1.1% in Astana). According to the component analysis, the growth in the number of breast cancer cases was due to a population increase (Δ_P_=+130.4% in Almaty and Δ_P_=+93.2% in Astana), with a notable decrease of factors related to the risk of getting sick (Δ_R_=−27.9% in Almaty, Δ_R_=−6.1% in Astana).

**Conclusion:**

This is the first epidemiological study to assess the changes in incidence and mortality from breast cancer in megapolises of Kazakhstan because of screening. The results of this study can be used to improve the government program to combat breast cancer.

## Introduction

Breast cancer is the most common malignant disease among the female population of Kazakhstan (1–3) like in many developed countries of the world (Canada, UK, US, Western Europe) (4–7). Breast cancer accounts for every 5^th^ tumor. About 4.6 thousand new cases and 1.3 thousand deaths are registered in Kazakhstan every year (8).

The analysis of incidence and mortality related to malignant neoplasms forms the basis for regional and national anti-cancer programs and is of paramount importance in justifying methods of disease prevention, measures for early diagnosis, and the development of screening programs (9–15). Breast cancer incidence and mortality rates differ in the countries with high and low risk, and some of these differences are related to the state of reporting and screening (11, 16–19).

Epidemiological studies of breast cancer allow defining the goals and objectives of disease prevention programs, including the planning of screening and diagnostic activities aimed at early detection of the disease, as well as to develop program efficiency indicators and evaluating program implementation results (15, 20).

The study of epidemiological features of incidence and mortality in large cities is of definite scientific and practical interest as the leading diagnostic and research centers that conduct active anti-cancer activities are concentrated in large cities.

We aimed to assess the epidemiological aspects of breast cancer incidence and mortality among Almaty and Astana (Now Nur-Sultan) residents in 2009–2018.

## Materials and Methods

Information was mainly obtained from the state registry of breast cancer cases (Form 7, Form 35 of the Ministry of Health of the Republic of Kazakhstan) and the breast cancer mortality statistics of the Statistics Committee of the Ministry of National Economy of the Republic of Kazakhstan (Table C51). Data for two major cities of Kazakhstan – Almaty and Astana – was included in the analysis.

A retrospective study (2009–2018) using modern descriptive and analytical methods of epidemiology (9, 21–24) was conducted to study the breast cancer incidence and mortality and calculate the extensive crude, age-specific, standardized rates of incidence and mortality (annual, annual average, errors) with a 95% confidence interval (95% CI).

Standardized indicators were calculated by the direct method (World standard) (25) following the relevant recommendations (26). The obtained dynamics series were analyzed by the method of least squares (alignment). Geometrical means were applied to calculate the average annual growth/decrease (T_gr/dec_, %). Component analysis according to Dvoyrin and Aksel (27) was used to analyze the changes in the number of breast cancer cases. Abbreviations used in the tables: AN – absolute number, AA – average age, CR – crude rate, ASR – age-standardized rate.

## Results

In 2009–2018, 40,097 new cases of breast cancer were registered in Kazakhstan, of them, 5,753 (14.3%) were detected in the city of Almaty and 2,188 (5.5%) in the city of Astana. For 10 years studied, 13,013 women have died from breast cancer, including 1,825 (14.0%) and 614 (4.7%) deaths in Almaty and Astana, respectively.

The average age of patients with breast cancer in Almaty was 58.1±0.2 years what was statistically significantly higher (*t*=7.21, *P*=0.00) than in Astana (55.5±0.3 years). At the same time, the average age of death in Almaty (61.2±0.3 years) was similar to Astana (61.6±1.1 years) and showed no statistically significant difference (*t*=0.9, *P*=0.93).

**Fig. 1:**
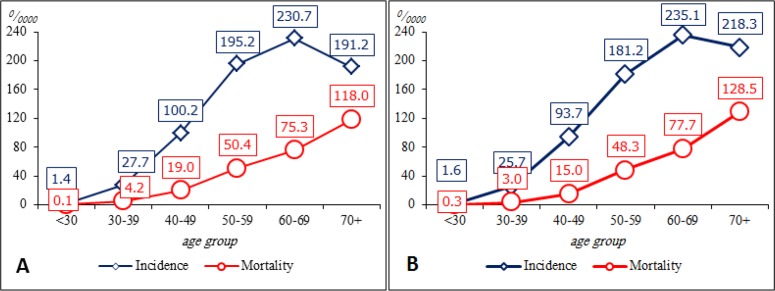
Age-specific breast cancer incidence and mortality among Almaty (A) and Astana (B), Kazakhstan residents, 2009–2018

The age-specific breast cancer incidence and mortality rates of in the cities studied were growing unimodally, with a peak of incidence at the age of 60–69 years and the peak of mortality at the age of 70 and above (Fig. 1).

The average annual crude incidence of breast cancer amounted to 68.2±3.2 00000 (95% CI=61.9–74.6) in Almaty and 52.4±2.1 00000 (95% CI=48.2–56.6) in Astana, with a statistically significant difference between the cities (*t*=4.13, *P*=0.000). The average crude mortality rates was 21.7±1.1 00000 (95% CI=19.6–23.8) in Almaty, and 14.9±0.7 00000 (95% CI=13.5–16.3) in Astana (t=5.22, *P*=0.000). Age-standardized rates were calculated to unify the results obtained and to eliminate the age difference. The calculated age-standardized breast cancer incidence and mortality rates are shown in Table 1. In Almaty, the age-standardized rates were found to be lower than the crude rates; in Astana, it was the opposite.

Certain trends were revealed when analyzing the age-specific incidence and mortality in large cities. The equalized incidence in Almaty in the age groups before 30, 30 to 39 and 40 to 49 years has decreased in the background of an increase in other age groups. In Astana, the incidence has increased in almost all age groups except 50–59 years (T_dec_=−2.1%), while the mortality has decreased almost in all age groups except for patients older than 60 years.

The above changes have generally affected the overall dynamics of crude incidence and mortality in the studied cities: of Almaty (T_gr_=+1.0 and T_dec_=−4.3, respectively) and Astana (T_gr_=+2.2 and T_dec_=−0.9, respectively). The same trends were observed in standardized incidence and mortality rates (Table 2). In the study period, the average annual morphological verification has amounted to 94.1±1.8% in Almaty and 97.8±0.8% in Astana (Table 3).

**Table 1:** Breast cancer incidence and mortality in Almaty and Astana, Kazakhstan 2009–2018

***Rates***		***The city of Almaty***			***The city of Astana***		

		***P±m***	***95% CI***	***T_gr/dec_***	***P±m***	***95% CI***	***T_gr/dec_***
Incidence	CR	68.2±3.2	61.9–74.6	+1.0	52.4±2.1	48.2–56.6	+0.3
	ASR	61.9±2.9	56.2–67.6	+0.8	61.2±2.3	56.7–65.7	+1.4
Mortality	CR	21.7±1.1	19.6–23.8	−3.8	14.9±0.7	13.5–16.3	−1.4
	ASR	19.2±1.0	17.3–21.1	−4.2	19.3±1.1	17.1–21.4	−1.1

**Table 2:** Average annual increase/decrease of equalized age-specific breast cancer incidence and mortality rates in Almaty and Astana, Kazakhstan 2009–2018

***Age group (yr)***	***The city of Almaty***		***The city of Astana***	

	***Incidence***	***Mortality***	***Incidence***	***Mortality***
<30	**−9.0**	**+3.3**	+1.0	−27.0
30–39	**−2.5**	−9.1	+0.3	−19.4
40–49	**−0.5**	−9.9	+1.9	−1.2
50–59	+1.6	−3.6	**−2.1**	−6.6
60–69	+1.3	−2.7	+4.6	**+0.2**
70+	+1.1	−3.7	+2.0	**+2.7**

**Table 3:** Dynamics of morphological verification (%) in Almaty and Astana, Kazakhstan 2009–2018

***City***	***2009***	***2010***	***2011***	***2012***	***2013***	***2014***	***2015***	***2016***	***2017***	***2018***	***P±m***
Almaty	90.1	87.7	94.1	97.4	86.6	88.9	98.6	99.2	98.7	99.8	94.1±1.8
Astana	98.2	94.6	96.8	94.8	100.0	96.8	99.2	99.6	99.2	99.0	97.8±0.7

**Fig. 2:**
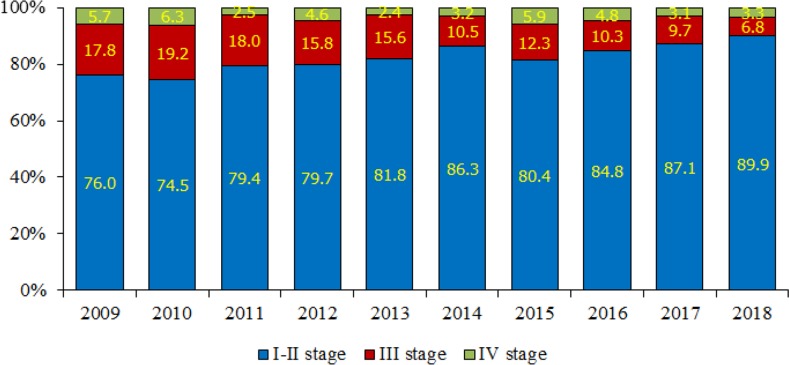
Staging of breast cancer patients in Almaty, Kazakhstan 2009–2018

**Fig. 3:**
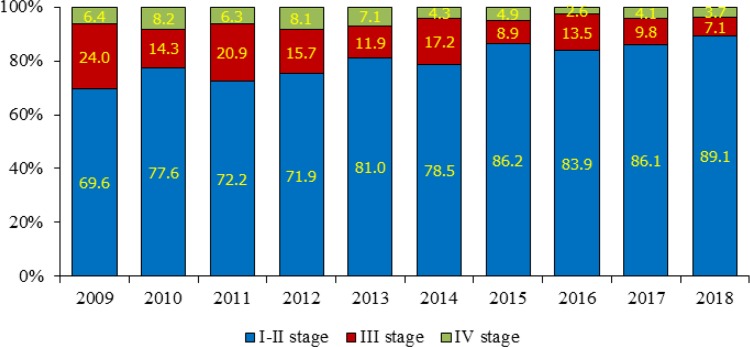
Staging of breast cancer patients in Astana, Kazakhstan 2009–2018

Figures 2 & 3 illustrate the staging of breast cancer patients in Almaty and Astana during the study years. The early breast cancer detection rate (the share of patients diagnosed with stage I-II cancer) in Almaty has increased from 76.0% (2009) to 89.9% (2018) (), with the average annual early detection rate of 82.0±1.7% (95% CI=78.7–85.2%). The share of patients diagnosed with stage III and IV cancer in Almaty has decreased from 23.5% (2009) to 10.1% (2018). The average annual shares of stage III and IV cancer in Almaty were equal to 13.6±1.4% (95% CI=10.8–16.4%) and 4.2±0.5% (95% CI=3.2–5.1%), respectively.

In Astana, the early detection rate has also increased from 69.6% (2009) to 89.1% (2018), with the average annual rate of 79.6±2.3% (95% CI=75.2–84.0%). The share of patients with stage III and IV cancer has notably decreased from 30.4% (2009) to 10.8% (2018). The average annual shares of stage III and IV cancer in Astana were equal to 14.3±1.8% (95% CI=10.9–17.8%) and 5.6±0.6% (95% CI=4.3–6.8%), respectively.

Crude breast cancer incidence rates for Almaty population has decreased from 62.8 00000 (2009) to 59.1 00000 (2018). At that, the average decrease has amounted to −3.6 00000 and was dependent on the changes in ASP (∑=Δ_A_=+1.1 00000 ), the disease risk (∑=Δ_R_=−4.4 00000 ), and the combined effect of changes in those parameters (∑=Δ_AR_=−0.3 00000 ).

In Astana, crude breast cancer incidence has increased from 54.4 00000 (2009) to 56.1 00000 (2018). The total increase (+1.7 00000 ) was associated with the changes in ASP (∑=Δ_A_=+4.8 00000 ) and the disease risk (∑=Δ_R_=−2.6 00000 ), while the combined effect of changes in those parameters has led to a decrease in crude incidence rates (∑=Δ_AR_=−0.5 00000 ).

The conducted component analysis revealed the factors that mainly influenced the changes in the number of patients with breast cancer in the studied cities:

1. Growth of population in large cities: Almaty – Δ_P_=+130.4%, Astana – Δ_P_=+93.2%;
2. Changes in the age structure of the population: Almaty – Δ_A_=+6.9%, Astana – Δ_A_=+11.2%.
3. The combined effect of changes in the number and age structure of the population: Almaty – Δ_PA_=+2.3%, Astana – Δ_PA_=+8.2%.
4. Change of disease risk: Almaty – Δ_R_=−27.9%, Astana – Δ_R_=−6.1%.
5. The combined effect of changes in the disease risk and the number of population: Almaty – Δ_PR_=−9.2%, Astana – Δ_PR_=−4.5%.
6. The combined effect of changes in the disease risk and the age structure of the population: Almaty – Δ_AR_=−1.9%, Astana – Δ_AR_=−1.1%.
7. The combined effect of changes in the disease risk and the age structure of the population: Almaty – Δ_PAR_=−0.6%, Astana – Δ_PAR_=−0.8%.

Thus, the prevalence of breast cancer is growing in large cities due to the overall population growth and changes in the age structure of the population.

## Discussion

In the study period, about 20% of new cases and deaths from breast cancer were registered in the two large cities of Kazakhstan (1, 2). These cities host more medical organizations than other regions of the country; therefore, the changes in epidemiological indicators allow assessing the efficiency of national mammalogical screening in the country, also in the megapolises.

Breast cancer prevails in the structure of malignancies in Kazakhstan women as has been shown before (1–3). It remains first in structure all over the country and in the national megapolises thus reflecting the global picture (5). At that, the share of patients with breast cancer was high in the studied cities at the age of 50–59 years what is similar to Kemalpas (28), Mumbai (29), and Bangkok (30). The average age of patients with breast cancer was statistically significantly higher in Almaty than in Astana. It might be related to the demographic difference in the age structure of the population as Almaty population belongs to a regressive type in comparison to Astana which is closer to a progressive type of population. (In Almaty, the age group below 15 years (the average of 18% in 2009–2018) is smaller than the age group of 50 years and above (25%). This indicates depopulation, is caused by a decrease in the birth rate and an increase in the overall mortality rate due to the relative prevalence of the older age groups. At the same time, in Astana, 23% of the population is below 15 years vs. 17% in the age group of 50 years and above).

Regarding deaths from breast cancer, a high share of deaths from breast cancer in Almaty was registered in the age group of 70 years and above (35.6%), while in Astana a high share of deaths from breast cancer was registered in the age group of 50–59 years and 70 years and above (29.0% each). Still, the difference in the average age of deaths from breast cancer in the studied cities (63.3 years in Almaty and 61.6 years in Astana) was not statistically significant. At that, the age-specific incidence was growing unimodally in both studied cities, with a peak at the age of 60–69 years. The mortality was also increasing, with a peak at the age of 70 and above. The obtained data corresponds to the results of many worldwide and Asian studies (5, 31–33).

In terms of incidence and mortality, Almaty and Astana have average rates of breast cancer incidence and mortality, which are comparable to such European and Asian countries (7) as Latvia (62.8 00000 ), Bulgaria (59.1 00000 ), Estonia (61.2 00000 ), Poland (59.1 00000 ), Singapore (64.0 00000 ), Korea (59.8 00000 ), and Japan (57.6 00000 ).

In Almaty, the age-standardized incidence rate was lower than the crude rates; in Astana, it was the opposite. It was due to a different age structure of population in the studied cities, as in Astana the population was generally younger, as mentioned above.

The breast cancer incidence in Astana in all age groups and in general during the study period was increasing except for the age of 50–59 years (T_dec_=−2.1%). This could be especially alarming taking into account the national screening conducted in 2012–2016 and addressed that very age group, i.e. people above 50 years. The increase in incidence observed in this group is characteristic of many countries implementing breast cancer screening (10).

In Almaty, we should note an increase of mortality among people under 30 years (T_gr_=+3.3%), while in other age groups the mortality has decreased. Moreover, in Astana, there was the highest decrease in mortality in this age group (T_dec_=−27.0%) and at the age of 30–39 years (T_dec_=−19.4%). In Astana, the mortality has increased in people above 60 years (60–69 years – T_gr_=+0.2, and 70+ – T_gr_=+2.7%). The literature sources do not report similar trends in the countries being screened. The average annual increase of equalized breast cancer incidence and mortality rates in Almaty and Astana is much less than in other major metropolises like New York (USA), Moscow (Russia) because their population is much bigger. However, in Shanghai (China) which has a higher population, the annual increase of breast cancer incidence is lower than in Kazakhstan.

Literature sources mention many factors associated with the etiopathogenesis of breast cancer. These factors can be grouped as follows: genetic, hormonal, reproductive, morphological factors, nutrition, lifestyle, ecology, previous pathology of the mammary glands, as well as ethnic and racial affiliation (15, 34–36).

Thus, the burden of breast cancer in large cities of Kazakhstan increases from year to year, as in other cities in the developed and developing countries of the world (37, 38). At the same time, the increase in life expectancy, the growth of urbanization and the influence of the Western lifestyle are the main causative factors that increase the risk of developing breast cancer (19, 39, 40).

## Conclusion

The study of breast cancer reveals a wide range of problems related to early detection and screening. The obtained results require further detailed investigation. Epidemiological studies of breast cancer incidence and mortality in Almaty and Astana highlight certain differences and peculiarities. The monitoring and evaluation of breast cancer indicators for 2009–2018 raise such questions as to why the incidence is decreasing and the mortality increasing in some age groups covered by screening. Still, there is some positive dynamics: a decrease in mortality (especially, in Almaty), as well as an increase in early detection rates. The analysis of early detection and neglect rates shows a positive increase in the share of patients with stage I-II cancer and a decrease in the share of patients with stage III-IV cancer in both Almaty and Astana. Changes in epidemiological indicators are undoubtedly the results of the impact of anticancer measures – the implementation of breast screening in the Republic. The obtained results can be used to improve anti-cancer activity in the country. Further study of screening efficiency is a priority for our continued research.

## Ethical considerations

Ethical issues (Including plagiarism, informed consent, misconduct, data fabrication and/or falsification, double publication and/or submission, redundancy, etc.) have been completely observed by the authors.
